# Non-redundant functions of two proline dehydrogenase isoforms in Arabidopsis

**DOI:** 10.1186/1471-2229-10-70

**Published:** 2010-04-19

**Authors:** Dietmar Funck, Sonja Eckard, Gudrun Müller

**Affiliations:** 1 Department of Plant Physiology and Biochemistry, Biology Section, University of Konstanz, Universitätsstrasse 10, 78464 Konstanz, Germany

## Abstract

**Background:**

Proline (Pro) accumulation is a widespread response of prokaryotic and eukaryotic cells subjected to osmotic stress or dehydration. When the cells are released from stress, Pro is degraded to glutamate by Pro-dehydrogenase (ProDH) and Pyrroline-5-carboxylate dehydrogenase (P5CDH), which are both mitochondrial enzymes in eukaryotes. While *P5CDH *is a single copy gene in Arabidopsis, two *ProDH *genes have been identified in the genome. Until now, only *ProDH1 *(At3g30775) had been functionally characterised.

**Results:**

We demonstrate vasculature specific expression of the Arabidopsis *ProDH2 *gene (At5g38710) as well as enzymatic activity and mitochondrial localisation of the encoded protein. Expression levels of *ProDH2 *are generally low, but increased in senescent leaves and in the abscission zone of floral organs. While sucrose represses *ProDH2 *expression, Pro and NaCl were identified as inducers. Endogenous *ProDH2 *expression was not able to overcome Pro sensitivity of *ProDH1 *mutants, but overexpression of a GFP-tagged form of ProDH2 enabled the utilisation of Pro as single nitrogen source for growth. Amongst two intronic insertion mutants, one was identified as a null allele, whereas the other still produced native *ProDH2 *transcripts.

**Conclusions:**

Arabidopsis possesses two functional ProDHs, which have non-redundant, although partially overlapping physiological functions. The two ProDH isoforms differ with respect to spatial, developmental and environmental regulation of expression. While *ProDH1 *appears to be the dominant isoform under most conditions and in most tissues, *ProDH2 *was specifically upregulated during salt stress, when *ProDH1 *was repressed. The characterisation of *ProDH2 *as a functional gene requires a careful re-analysis of mutants with a deletion of *ProDH1*, which were so far considered to be devoid of ProDH activity. We hypothesise that ProDH2 plays an important role in Pro homeostasis in the vasculature, especially under stress conditions that promote Pro accumulation.

## Background

Arabidopsis, like most plant species, accumulates free proline (Pro) to high concentration in response to salt and drought stress. The physiological function of Pro is subject to controversial debate, and Arabidopsis plants with a reduced capacity to accumulate Pro showed only a moderate decrease or no change in stress tolerance [1-3]. Pro is suggested to act as a compatible osmolyte, a stabilising agent for macromolecules, a radical scavenger or as a nitrogen and energy store for the recovery phase [4,5]. Other hypotheses attribute the beneficial function to the process of Pro metabolism rather than the mere accumulation of Pro. Along this line, energy transfer between cellular compartments, regeneration of electron acceptors or signalling functions have been proposed [6,7].

**Figure 1:**
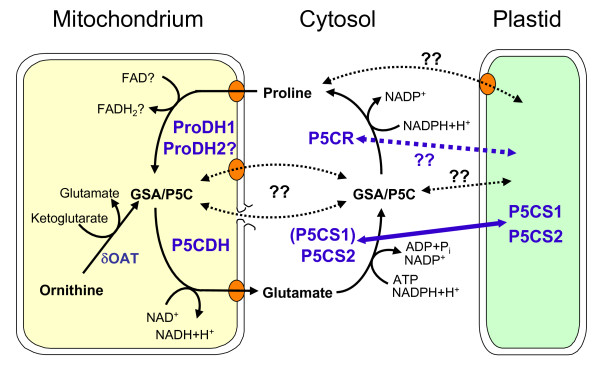
**Proline metabolism in Arabidopsis**. Schematic illustration of the current knowledge on Pro metabolism and its intracellular distribution. Black arrows indicate metabolic fluxes, enzymes are given in bold blue letters, for abbreviations see the main text. The molecular identity of mitochondrial and plastidic Pro and glutamate transporters is currently not known. Alternating localisation between cytosol and plastids was demonstrated for P5CS1 and P5CS2, the localisation of P5CR requires further detailed analysis. Export or leakage of GSA/P5C from mitochondria was postulated but experimental evidence is lacking.

The biochemistry of Pro biosynthesis and degradation has been intensively studied and most of the participating proteins and the genes encoding for these enzymes were characterised with respect to temporal and spatial expression patterns and enzymatic properties (Fig. 1; [5]). The first step of Pro synthesis is catalysed by Pyrroline-5-carboxylate synthetase (P5CS), which uses glutamate, NADPH and ATP to generate glutamate-γ-semialdehyde (GSA). GSA is in spontaneous equilibrium with the cyclic pyrroline-5-carboxylate (P5C), which is converted to Pro by P5C reductase (P5CR), again consuming NADPH. Arabidopsis contains two isoforms of P5CS that are differentially regulated. *P5CS2 *(At3g55610) expression was consistent with a housekeeping function, and the P5CS2 protein was found to be localised in the cytosol under normal conditions while being partially re-located to plastids during stress [3,8,9]. Pro accumulation under stress conditions is primarily contingent upon induction of *P5CS1 *(At2g39800) expression. Also P5CS1 was observed in the cytosol, partly as presumably inactive aggregates, and re-localised to plastids during stress [3]. P5CR seems to be predominantly localised in the cytosol, while some activity was also associated with plastid preparations [10,11]. However, direct investigations of the localisation and enzymatic properties of Arabidopsis P5CR (At5g14800) are still pending. A second pathway for Pro synthesis from ornithine had also been postulated, but characterisation of Ornithine-δ-aminotransferase indicated that ornithine is degraded to glutamate inside the mitochondria prior to its conversion to Pro by the standard pathway [12,13]. Loss or gain of function mutants provided valuable evidence for the physiological role of Pro accumulation, although the interpretation of the mutant phenotypes was always hampered by the primary function of Pro in protein biosynthesis [4].

For degradation, Pro is imported into the mitochondria, where it is converted back to glutamate by the tightly coupled activities of Pro dehydrogenase (ProDH) and P5C dehydrogenase (P5CDH [14,15]). In Arabidopsis, one ProDH (*ProDH1*; At3g30775) and one P5CDH (At5g62530) have been characterised at the molecular level. *ProDH1 *and *P5CDH *expression is repressed by osmotic stress and upregulated by Pro, with the changes being more pronounced for *ProDH1 *[4]. Also in flowers, strong expression of *ProDH1 *was observed in the stigma and in pollen, while *P5CDH *is only upregulated in pollen [16,17]. Mutants with defects in Pro degradation grew without obvious phenotypic differences under greenhouse conditions, only a slight decrease in seed quality was noted for *p5cdh *mutants [16,18-20]. Common to the *p5cdh *mutants and the *ProDH1 *mutant *pdh1-1 *were an enhanced and prolonged accumulation of Pro during stress and an unexpected hypersensitivity to external Pro supply in the absence of stress. In wildtype plants, external application of harmful amounts of Pro caused the loss of mitochondrial and plastid integrity [21]. Hare *et al*. (2002) hypothesised that excess Pro degradation would cause misdirection of electrons to O_2 _in mitochondria and plastids due to electron overflow or acceptor limitation, respectively. Recent results confirmed the Pro-dependent production of reactive oxygen species (ROS) in mitochondria, an effect that was more pronounced in *ProDH *overexpressing or *p5cdh *mutant plants [22].

The only obvious gap in the molecular characterisation of Pro metabolism in Arabidopsis remains a second isoform of ProDH (*ProDH2*, At5g38710), which has been identified as an expressed gene with high homology to *ProDH1 *[1,23]. The predicted pre-proteins of ProDH1 and ProDH2 share 75% identical amino acids (aa). Hanson *et al*. (2008) demonstrated a direct induction of *ProDH2 *by the sucrose-repressed transcription factor bZIP11 [24]. However, until now the enzymatic functionality of ProDH2 had not been addressed. Here, we demonstrate that ProDH2 expressed in yeast can mediate Pro degradation equal to ProDH1. Overexpression of a ProDH2-GFP construct in Arabidopsis was able to rescue the Pro-hypersensitive phenotype of the *pdh1-1 *knockout mutant. We detected ProDH2-GFP in the mitochondria and analysis of GUS expression under control of the *ProDH2 *promoter demonstrated specific expression in the vascular tissue and in the abscission zone of petals, sepals and stamina. In striking contrast to *ProDH1*, *ProDH2 *expression was induced by salt stress and was absent in reproductive tissues.

## Results

### At5g38710 encodes a protein with ProDH activity

**Figure 2:**
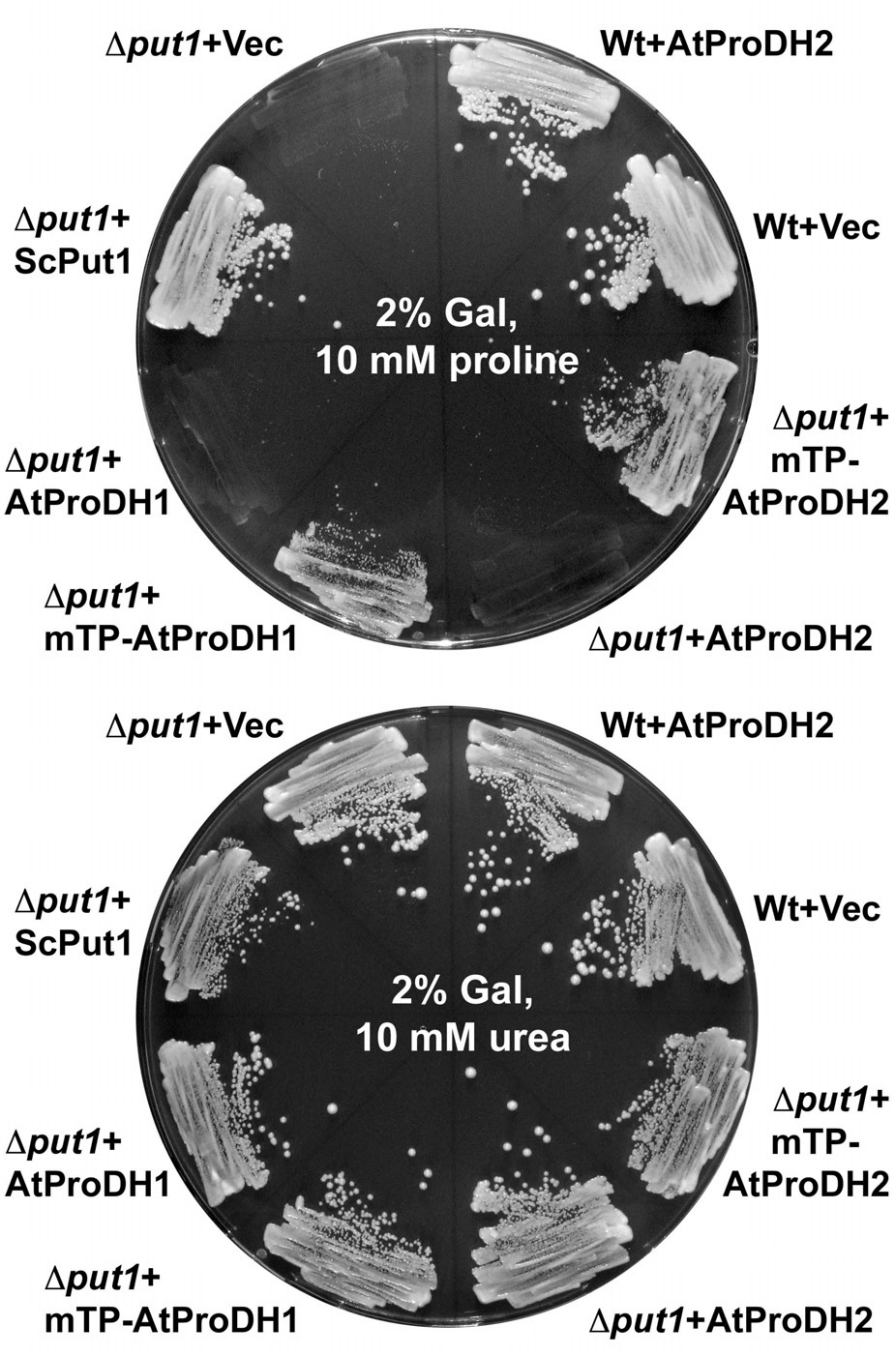
**ProDH2 expression complements a yeast Δ*put1 *mutant**. Yeast wildtype or Δ*put1 *mutant with various ProDH expression constructs were grown for 4 d on minimal medium with 2% (w/v) galactose (Gal) as inducing carbon source and 10 mM Pro (upper plate) or 10 mM urea (lower plate) as the sole nitrogen source. Expression of ScPut1 confers wildtype growth to the Δ*put1 *mutant. Native AtProDH pre-proteins do not complement the Pro utilisation deficiency of the Δ*put1 *mutant. Replacement of the predicted Arabidopsis mTPs by the mTP of the yeast *Sdh1 *gene confers functional expression and the capability to metabolise external Pro as N-source (mTP-AtProDH1 and mTP-AtProDH2).

Reports on successful determination of ProDH activity from plant mitochondrial membranes are very rare. A variety of papers ascribe a soluble activity of Pro dependent NAD^+ ^reduction at pH 10.3 to ProDH, but this is more likely to be the reverse reaction of P5CR (Giuseppe Forlani, unpublished results). Therefore we choose to test the enzymatic function of the putative ProDH2 (At5g38710) by heterologous expression in yeast. To obtain a suitable test system, we generated a mutant yeast strain in which the *Put1 *gene, encoding the single ProDH, was replaced by a kanamycin and geneticin resistance cassette by homologous recombination. The resulting geneticin resistant strain Δ*put1 *was unable to catabolise Pro as a source of nitrogen for growth (Fig. 2). Transformation of the Δ*put1 *strain with a plasmid harbouring the full-length cDNA of At5g38710 did not enable the cells to metabolise Pro. A similar observation for ProDH1 has been described by Kiyosue *et al*. (1996) and was confirmed by our results [25]. Exchange of the predicted mitochondrial transit peptide (mTP) of the putative ProDH2 (aa 1-29) against the mTP of the flavoprotein subunit of yeast succinate dehydrogenase (*ScSdh1*) lead to the expression of a protein that rescued the Pro metabolism defect of the Δ*put1 *strain. Also an exchange of the first 39 amino acid residues of ProDH1 against the *ScSdh1*-mTP enabled the expression of a functional protein. The *ScSdh1*-mTP was chosen because it is unlikely to interfere with the functionality test for the Arabidopsis ProDHs. From these results we concluded that the name *ProDH2 *was justified for At5g38710. Under non-inducing conditions, with glucose instead of galactose as carbon source, none of the *ProDH1 *or *ProDH2 *expression constructs enabled the Δ*put1 *strain to catabolise Pro (data not shown). Expression of the full-length *ProDH2 *cDNA in wildtype yeast did not alter the growth properties and western blot analysis of cells expressing a ProDH2 protein with 6×His-tag confirmed the presence of the recombinant protein (Fig. 2 and data not shown). These findings indicate that the native ProDH2 protein is not harmful to yeast, but does not have sufficient ProDH activity to enable utilisation of Pro as nitrogen source.

### *ProDH2 *is expressed specifically in the vascular tissue

**Figure 3:**
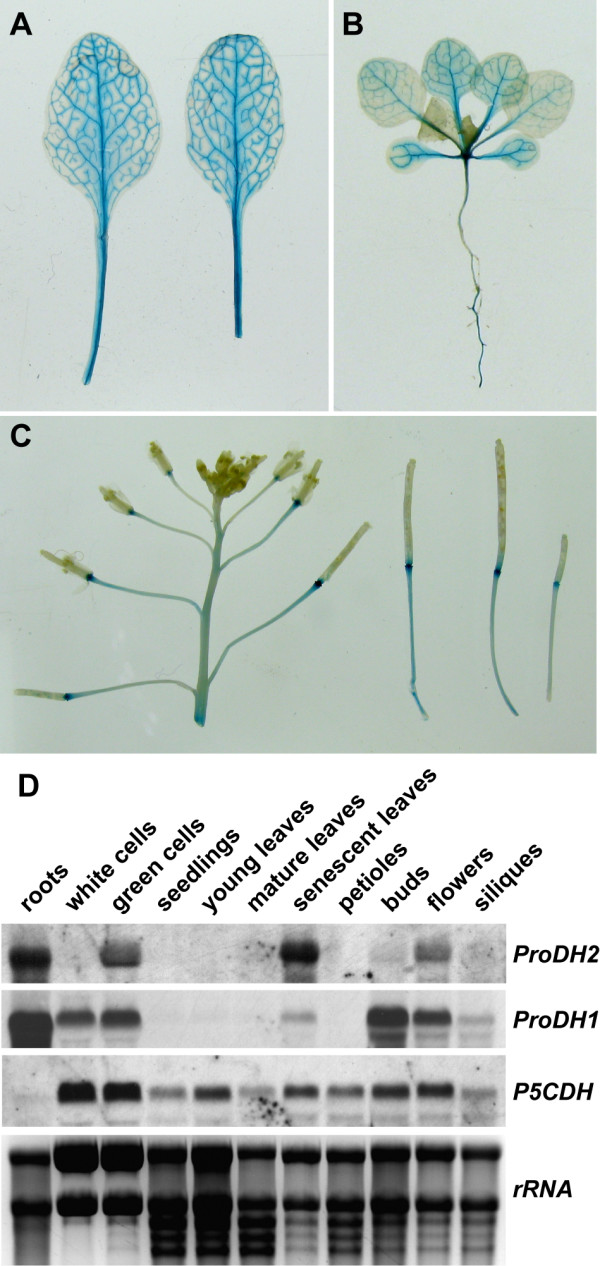
**Tissue distribution of *ProDH2 *expression**. A-C: *ProDH2*-promoter activity visualised in transgenic plants carrying a *ProDH2*-promoter-*Gus *fusion construct. Tissues were stained for 6 h in 1 mM X-Gluc and destained in 80% (v/v) EtOH. A: Two mature leaves of a 6-week-old plant. B: Three-week-old seedling showing enhanced staining with progressing leaf age. C: Inflorescence and young siliques showing GUS activity in the abscission zone between the pistils and the floral stalk. D: Northern blot analysis of transcript levels of Pro degradation genes in various tissues and two different cell cultures. The picture of the EtBr stained gel is shown to demonstrate RNA integrity and equal loading.

The presence of two *ProDH *genes in the Arabidopsis genome, which both encode functional ProDHs, raised the question of the specific function of ProDH2. Expression analysis of *ProDH1 *indicated ubiquitous expression in all organs, with the highest expression levels detected in pollen grains and in the stigma of the carpel [17]. To compare the expression of *ProDH1 *and *ProDH2*, we fused 1500 bp of upstream sequence of the *ProDH2 *gene to a β-glucuronidase (GUS) gene. Bioinformatic analysis of 3000 bp of genomic sequence upstream of the start codon of *ProDH2 *with the Athena analysis tool did not reveal any potential transcription factor binding sites that were more abundant than the expectations for random distribution would predict [26,27]. However, consensus motifs for an abscisic acid responsive element and a drought responsive element were identified at -880 and -1395 bp, respectively. Most of the putative binding motifs that were found between -1500 and -3000 bp were also present in the region between -1 and -1500 bp. Therefore we chose to include only the region up to -1500 bp in the GUS-construct. Transgenic plants harbouring this construct expressed GUS activity exclusively in the vascular tissue (Fig. 3A-C). Of the 30 analysed primary transformants, all showed a very similar expression pattern, albeit the amount of GUS activity detected by X-Gluc staining varied considerably (Data not shown). Staining was generally weak in the youngest leaves and strongest in the older leaves. For further analysis, plants with intermediate activity in mature leaves were chosen. In inflorescences, the *ProDH2*-promoter induced GUS expression only in the abscission zone immediately below the pistils or siliques. This high expression persisted through silique maturation, demonstrating a partial spatial separation of *ProDH1 *and *ProDH2 *expression in flowers. Northern blot analysis of RNA isolated from various tissues of soil grown Arabidopsis wildtype plants confirmed that the expression of *ProDH2 *is stronger in floral organs (Fig. 3D). In young (less than 7 mm long) and mature leaves (more that 3 cm long), no *ProDH2 *transcripts were detected, presumably due to the low relative content of vascular tissue, whereas a strong signal in senescent leaves (showing anthocyanin accumulation and visible reduction in chlorophyll content) confirmed age-dependent regulation of *ProDH2 *expression. In petioles from mature leaves, no *ProDH2 *expression was detected by northern blot although this part of the leaf should be enriched in vascular tissue. Also in roots and leaf-derived cell cultures, a strong expression of *ProDH2 *was detected. In contrast, *ProDH1 *was expressed also in a root-derived cell culture and was only slightly induced in senescent leaves. In the inflorescence, *ProDH1 *expression reached its peak before *ProDH2*. *P5CDH *was detected in all organs analysed. However, a surprisingly low transcript level of *P5CDH *was observed in roots, where both *ProDH *isoforms were strongly expressed.

### *ProDH2 *is induced by proline and salt but repressed by sugar

**Figure 4:**
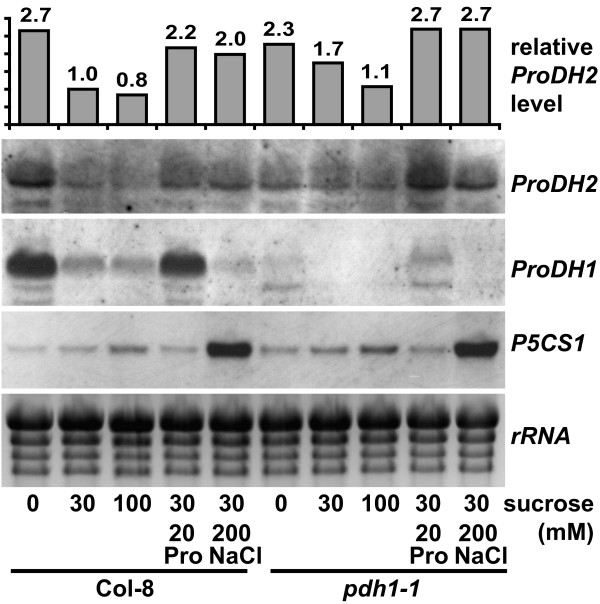
***ProDH2 *is induced by Pro and repressed by sucrose**. Three-week-old wildtype (Col-8) or *ProDH1*-knockout mutant (*pdh1-1*) seedlings from sterile culture were transferred for 6 h to liquid half-strength MS medium with the indicated supplements prior to RNA extraction and northern blot analysis. The same membrane was hybridised consecutively with specific probes for *ProDH2*, *P5CS1 *and *ProDH1*. During the detection of *P5CS1*, no remainders of the *ProDH2 *specific signal were observed. The histogram above the northern blot panel indicates the intensity of the *ProDH2*-specific signal normalised to the level of Col-8 in medium with 30 mM sucrose.

Since the variation of GUS activity between different transgenic lines was high, we used northern blot analysis to investigate the regulation of *ProDH2 *expression. Hanson *et al*. (2008) identified *ProDH2 *as a direct target of the transcription factor bZIP11 [24]. In agreement with these findings, *ProDH2 *transcript levels were higher when plants were incubated in medium without sucrose compared to treatment with 30 or 100 mM sucrose (Fig. 4). Also incubation for 6 h in medium containing 20 mM Pro or 200 mM NaCl in addition to 30 mM sucrose enhanced *ProDH2 *expression slightly. These concentrations were found to be sub-lethal during the period of the treatment and effective in triggering changes in Pro metabolism. Induction of *ProDH2 *by salt stress was surprising, since *ProDH1 *is repressed under these conditions. Induction of *P5CS1 *expression by 200 mM NaCl demonstrated that the treatment was efficient in triggering Pro accumulation. In general, the signals obtained for *ProDH2 *were near the detection limit, while *ProDH1 *detection yielded much stronger signals. This observation was confirmed by analysis of publicly available microarray data, in which *ProDH2 *expression is frequently not clearly detected (data not shown [28,29]). For example, in the AtGenExpress stress series, the maximal signal intensity for *ProDH1 *is 7.7-fold higher than for *ProDH2*. In the same dataset, the *ProDH1 *expression pattern was more similar to *P5CDH *than to *ProDH2*. Pathogen challenge and drought stress were among the rare cases, in which microarray analyses detected a stronger expression of *ProDH2 *than of *ProDH1*.

We also analysed potential compensatory changes of *ProDH2 *expression in the *ProDH1 *knockout mutant *pdh1-1 *[20]. Basal levels and sugar-dependent regulation of *ProDH2 *were only mildly affected in the *pdh1-1 *mutant. Induction of *ProDH2 *by Pro was slightly enhanced, presumably by the higher intracellular Pro content caused by lack of the ProDH1 isoform, which seems to be predominant in most tissues of wildtype plants. *P5CS1 *transcript levels were the same in wildtype and *pdh1-1 *mutant plants, while *ProDH1 *transcripts were mostly undetectable in the mutant. Under conditions of *ProDH1 *induction, some aberrant transcripts were also detected in *pdh1-1*.

### A role of ProDH2 in Pro tolerance

**Figure 5:**
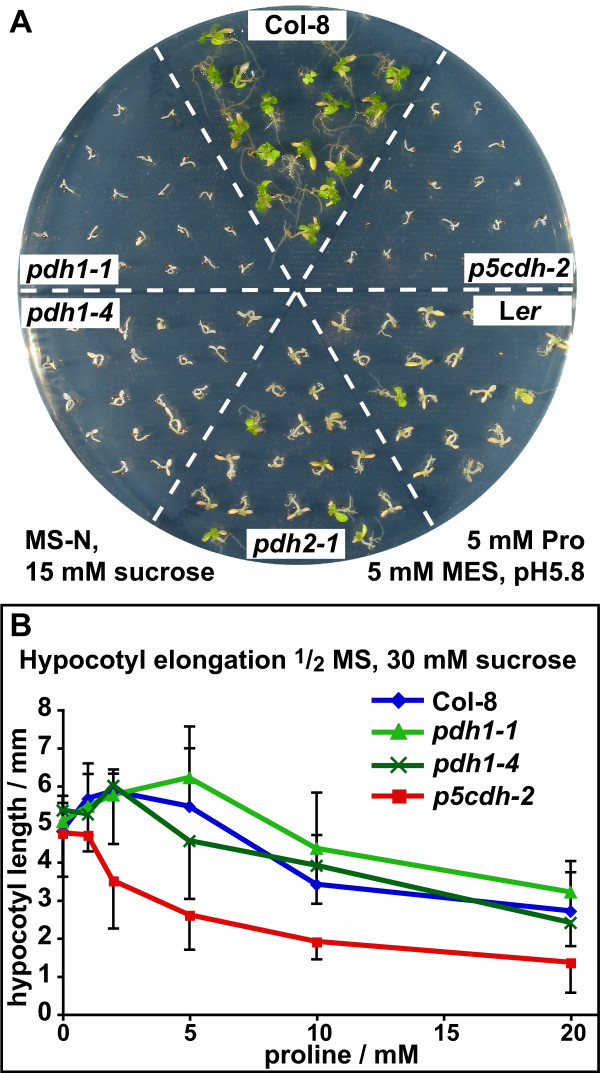
***p5cdh-2 *mutants are more sensitive to external Pro supply than *pdh1 *mutants**. A: Col-8 and L*er *wildtypes, *pdh1-1*, *pdh1-4*, *pdh2-1 *and *p5cdh-2 *seedlings were cultivated for 2 weeks on MS medium with 5 mM Pro as the sole source of nitrogen and 15 mM sucrose as carbon source, buffered to pH 5.8 with 5 mM MES. B: Hypocotyl elongation of Col-8 wildtype, *pdh1-1*, *pdh1-4 *and *p5cdh-2 *seedlings cultivated on MS medium supplemented with 30 mM sucrose and varying concentrations of Pro. Error bars indicate SD of ≥ 10 seedlings per genotype. The experiments were repeated with similar results.

The low and spatially limited expression of *ProDH2 *posed the question of whether the ProDH2 protein can make a significant contribution to Pro metabolism. Therefore we directly compared the Pro sensitivity of *pdh1-1*, *pdh1-4 *(see below) and *p5cdh-2 *mutants, reasoning that Pro degradation is completely blocked in *p5cdh-2 *mutants, while *pdh1 *mutants still contain the active ProDH2 isoform. As described earlier, neither of these mutants was able to use 5 mM Pro as the sole source of nitrogen (Fig. 5A). Seedlings of both mutant genotypes did not expand properly and stayed etiolated, while wildtype seedlings turned green and developed true leaves. Compared to Col-8, the L*er *ecotype showed a decreased ability to de-etiolate and grow with Pro as the only nitrogen source. A transposon insertion mutant devoid of *ProDH2 *(*pdh2-1*; see below) did not show increased sensitivity compared to the parental L*er *ecotype.

When *pdh1 *and *p5cdh *mutants were assayed for Pro sensitivity in the presence of mineral nitrogen, only *p5cdh-2 *mutants showed a dose dependent inhibitory effect of Pro on hypocotyl elongation (cell elongation) and fresh weight accumulation (cell growth) that was different from the wildtype (Fig 5B and data not shown). These findings indicate that expression of ProDH2 is not sufficient to compensate fully for the lack of ProDH1, but may still be able to confer limited Pro tolerance in the presence of mineral nitrogen. Contrary to the loss of *ProDH1 *or *P5CDH *expression, loss of *ProDH2 *did not result in Pro hypersensitivity.

### Robust splicing of T-DNAs in the first intron of *ProDH1 *and *ProDH2*

**Figure 6:**
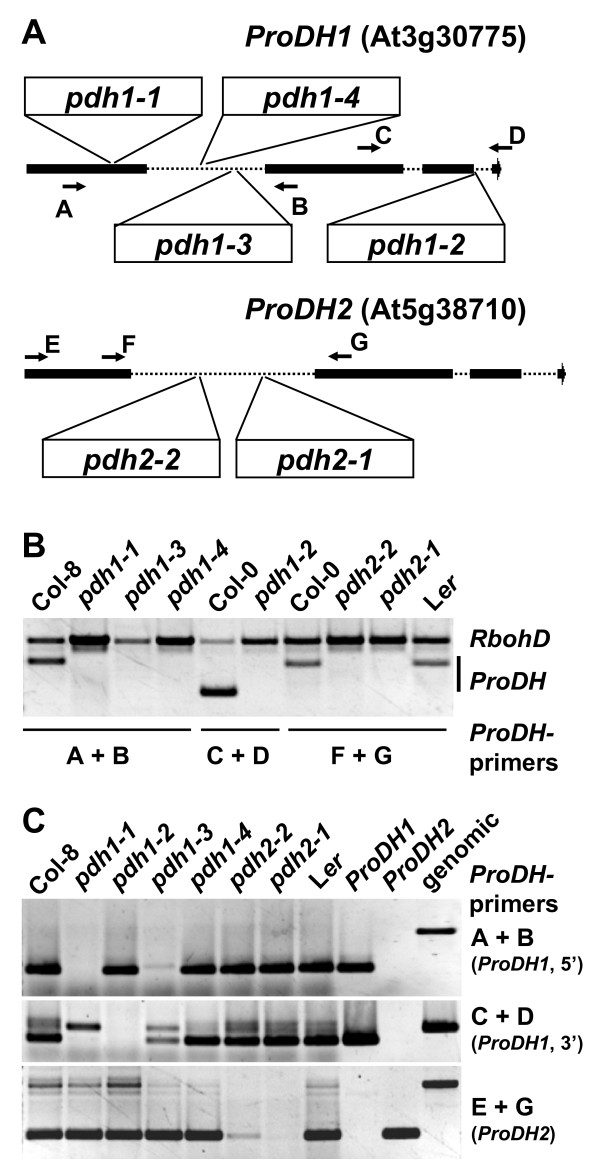
**Knockout lines for *ProDH1 *and *ProDH2 *and T-DNA excision during splicing**. A: Graphic representation of the exon-intron structure of the *ProDH1 *and *ProDH2 *genes with the position of T-DNA insertions in the analysed mutant lines (see material and methods section). Arrows with letters indicate the binding sites of primers used for PCR analyses in B and C. B: PCR on genomic DNA with two primer pairs, one for *RbohD *(At5g47910 as internal control, fragment size 1272 bp) and one *ProDH1 *or *ProDH2 *specific primer pair. Absence of *ProDH *specific PCR products demonstrates that all mutant plants were homozygous for the respective T-DNA insertion. C: RT-PCR analysis of *ProDH1 *and *ProDH2 *expression in the mutant lines demonstrates the presence of native transcripts in *pdh1-4 *and *pdh2-2*. PCR reactions with cloned cDNAs and genomic DNA (leftmost three lanes) demonstrate specificity of the PCR products. cDNAs for C and genomic DNA for B were obtained from the same samples, a slight contamination of the RNA samples with genomic DNA caused the additional amplification of intron-containing PCR products in the RT-PCRs.

To enable a direct comparison of mutants completely devoid of ProDH activity to the *p5cdh-2 *mutant, we screened the public T-DNA insertion lines for *pdh2 *mutants. Several lines (SALK_108179, GABI_918D08, SAIL_90_G05, GABI_187C05) with T-DNA insertions immediately upstream or downstream of the *ProDH2 *coding sequence had little or no effect on transcript abundance (data not shown). Two lines were identified with a T-DNA or a transposon in the first intron of *ProDH2 *(GABI_328G05 and GT1788, respectively). By northern blot and RT-PCR analysis we were unable to detect native transcripts in homozygous GT1788 plants, while both aberrant and a small amount of native transcripts were present in GABI_328G05 (Fig. 6 and data not shown). Therefore we termed these two insertion-lines *pdh2-1 *and *pdh2-2*, respectively. Histochemical analysis revealed no detectable GUS activity in *pdh2-1 *plants, indicating that the *GUS *gene of gene-trap construct contained in the transposon is not in frame with the first *ProDH2 *exon after potential splicing events (data not shown). A similar analysis of *ProDH1 *insertion lines confirmed the absence of native transcripts in *pdh1-1*, SALK_081276 (*pdh1-2*) and GABI_308F08 (*pdh1-3*). In line SALK_119334 (*pdh1-4*), which carries an inverted tandem repeat of the T-DNA in the first intron of *ProDH1*, a mixture of aberrant and native transcripts was detected, similar to the *pdh2-2 *insertion line. When grown under greenhouse conditions, all *ProDH1 *or *ProDH2 *insertion lines showed no phenotypic differences to the corresponding wildtypes (data not shown). The production of useful *pdh1*/*pdh2 *double mutants is impeded by the fact that the insertion line *pdh2-1 *is in L*er *background, in which stress-responsive Pro accumulation and Pro sensitivity differs from Col-8 (Fig. 5A and data not shown). Therefore, double mutants will have to be compared to multiple families of Col-8/L*er *hybrids or crossed several times to one of the parental ecotypes.

### ProDH2 is localised to mitochondria

**Figure 7:**
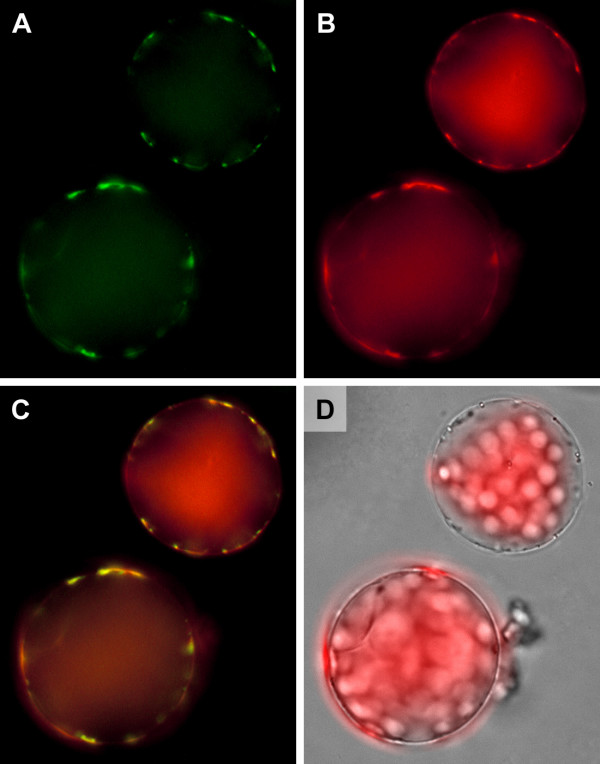
**Subcellular localisation of ProDH2**. False colour images of protoplasts isolated from Arabidopsis plants stably transformed with a 35S:*ProDH2:GFP *fusion construct and stained with MitoTracker Orange. A: GFP fluorescence depicted in green; B: MitoTracker fluorescence depicted in red; C: Overlay of A and B demonstrating co-localisation of ProDH2-GFP with mitochondria. D: Overlay of a transmitted light picture with chlorophyll autofluorescence.

The reason for the inability of *ProDH1 *mutants to utilise Pro as nitrogen source, despite a functional *ProDH2 *copy, could be that ProDH2 is not in the correct subcellular localisation to support Pro degradation. Prediction programs clearly indicated a mitochondrial localisation, which we analysed with the help of transgenic plants expressing a ProDH2-GFP fusion protein. The GFP signals obtained from such plants were much weaker compared to ProDH1-GFP expression driven by the same promoter, but both ProDH2-GFP and ProDH1-GFP were clearly localised in mitochondria (Fig. 7 and Additional File 1). Subcellular localisation and enzymatic functionality of ProDH2 indicated that the low expression level and/or the tissue specificity were responsible for the inability of *pdh1-1 *mutants to utilise Pro as nitrogen source.

### ProDH2-GFP overexpression rescues the *pdh1-1 *mutant

**Figure 8:**
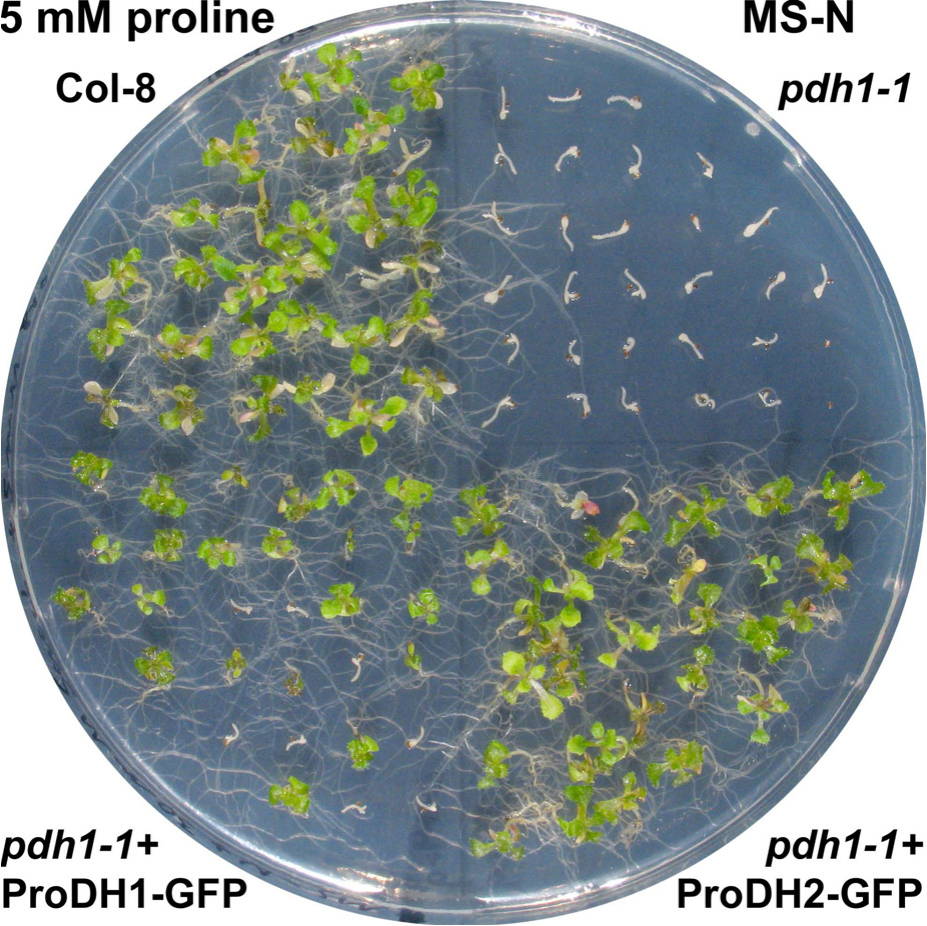
**ProDH-GFP expression rescues the *pdh1-1 *mutant**. Wildtype Col-8, *pdh1-1 *and *pdh1-1 *transformed with 35S:*ProDH1:GFP *or 35S:*ProDH2:GFP *were cultivated for 3 weeks on MS-N medium with 30 mM sucrose and 5 mM Pro as the only source of nitrogen. The transformants are segregating T_2 _populations, therefore not all seedlings are able to utilise Pro as nitrogen source.

To test the functional equivalence of ProDH2 and ProDH1, also the *pdh1-1 *mutant was transformed with the constructs for the 35S-driven expression of ProDH1-GFP and ProDH2-GFP. Overexpression of ProDH2-GFP or ProDH1-GFP restored the capability of *pdh1-1 *mutants to use Pro as the sole source of nitrogen (Fig. 8). These findings indicate that tissue specificity and expression levels are the main determinants of the different functionality of the two ProDH isoforms in Arabidopsis.

## Discussion

### Functional expression of Arabidopsis ProDHs in yeast

In the initial characterisation of ProDH1, two groups reported conflicting results concerning the expression of a functional protein from the native Arabidopsis gene in yeast. The *contra *group circumvented the problem by using the mTP of the yeast ProDH gene *Put1*, which might in itself interfere with the functionality test [25]. The data provided by the *pro *group lack proper positive and negative controls, for which it is impossible to tell if there is some strain specificity in the use of non-native mTPs [30]. Both studies suffer from the use of tryptophan auxotrophic strains, which necessitated the addition of low amounts of tryptophan, a potential nitrogen source. We circumvented these limitations by generating a Δ*put1 *deletion strain in the 23344c background, which is wildtype except for the *ura3 *mutation that served as a selection marker for the expression plasmids. In this background, the native cDNAs of either *ProDH *gene from Arabidopsis did not confer sufficient ProDH activity to enable Pro utilisation. Exchange of the predicted mTPs for the N-terminus of a metabolically unrelated mitochondrial protein from yeast allowed the expression of functional ProDHs as evidenced by complementation of the Pro utilisation defect of the Δ*put1 *strain. From this we concluded that both *ProDH *genes from Arabidopsis encode proteins that mediate Pro catabolism to P5C/GSA. Functional expression of the hybrid genes in yeast indicated that protein folding and co-factor insertion of the Arabidopsis proteins worked correctly in the heterologous system. Mitochondrial localisation of ProDH1-GFP and ProDH2-GFP fusion proteins in Arabidopsis demonstrated functionality of the ProDH-mTPs in the native organism. Complementation of the Pro hypersensitive phenotype of Arabidopsis *pdh1-1 *mutant by overexpression of ProDH1-GFP or ProDH2-GFP provided further evidence that both proteins have the same enzymatic activity. It remains to be investigated, why the native Arabidopsis *ProDH *genes do not enable the expression of sufficient amounts of active and correctly targeted protein, whereas the Arabidopsis *P5CDH *gene produced a functional protein with its native mTP [19].

### Spatial, temporal and metabolic regulation of *ProDH2 *expression

Analysis of the tissue specificity of *ProDH2 *expression by promoter-*Gus *fusion demonstrated that *ProDH2 *expression is restricted to the vascular tissue. This finding is in agreement with regulation of *ProDH2 *expression by the transcription factor bZIP11, which is also spatially confined to the vasculature [24,31]. Translation of bZIP11 is repressed by sucrose, which resulted in reduced transcript levels of *ProDH2 *in the presence of high sucrose concentrations. High sucrose concentrations (3%) might also be the reason for the absence of *ProDH2 *transcripts in the root-derived heterotrophic cell culture. The medium of the mixotrophic leaf-derived cell culture contained 1% sucrose (roughly corresponding to 30 mM), which is not sufficient to switch off *ProDH2 *expression. *ProDH2*-promoter driven *Gus *expression increased with leaf age and was strongest in the abscission zone of the floral organs, indicating that developmental signals also play a role in *ProDH2 *regulation. *ProDH1*, which had also enhanced promoter activity in the abscission zone, was additionally strongly expressed in pollen, in the stigma and in developing embryos, where no *ProDH2 *expression was detected [17]. In roots, both *ProDH1 *and *ProDH2 *were strongly detected, while *P5CDH *was only weakly expressed. Potentially, arginine catabolism, which is the second task of P5CDH, is less prominent in roots. Alternatively, the strong *ProDH *expression could result from the preparation of the roots by extensive washing in (hypotonic) tap water. Expression changes of *ProDH1 *are reportedly very fast, while regulation of *P5CDH *was slower [25,32]. Increased *ProDH2 *expression in old leaves was confirmed by northern blot analysis and evaluation of publicly available microarray data [28,29]. The spatial expression pattern was the most striking difference between *ProDH1 *and *ProDH2 *expression but also NaCl treatment caused down-regulation only of *ProDH1*. Similarly, Ribarits *et al*. (2007) found a differential regulation of two *ProDH *isoforms during dehydration in tobacco, while under these conditions the Arabidopsis *ProDHs *were co-regulated [23]. Over the broad range of different treatments documented in the microarray data collection, co-regulation of *ProDH1 *and *ProDH2 *is only sparsely observed, supporting our findings that the two ProDH isoforms fulfil at least partially different physiological functions. Up-regulation of *ProDH2 *expression during salt or drought stress might indicate a special need for proline degradation in the vasculature, while down-regulation of *ProDH1 *occurs in the remaining tissues and enables proline accumulation. These findings imply that proline accumulation might not be favourable in the vascular tissue and the continued degradation of proline may provide energy and glutamate for other metabolic pathways or for long distance transport.

### ProDH2 cannot fully compensate the lack of ProDH1

Loss-of-function mutants of *ProDH1 *were identified and characterised as sensitive to external Pro supply and heat treatment [20,33]. The existence and potential activity of *ProDH2 *was neglected in these studies. Mutants in *P5CDH*, which is a single copy gene in Arabidopsis, were completely unable to degrade Pro [16]. In a direct comparison of *pdh1*, *pdh2 *and *p5cdh *mutants, we observed that *pdh1 *and *p5cdh *mutants were unable to utilise Pro as the sole nitrogen source, whereas *pdh2 *mutants grew equally well as the corresponding wildtype. When *pdh1-1*, *pdh1-4 *and *p5cdh-2 *mutants were grown on medium containing mineral nitrogen, only *p5cdh-2 *was more sensitive to growth-inhibition by external Pro than the wildtype. Preliminary characterisation of the *pdh2-1 *mutant showed a slightly enhanced Pro accumulation in response to salt stress but not after external Pro application (data not shown). Pro sensitivity and stress induced Pro accumulation differed between the Col-8 and L*er *wildtypes, confirming that a useful double mutant can only be produced after extensive backcrossing. From these data we conclude that the slightly enhanced *ProDH2 *expression in Pro treated *pdh1 *mutants could not fully compensate the loss of ProDH1, while it may be able to diminish the toxic effects of Pro. Alternatively, the toxic effects of Pro supply might result from P5C, which is only expected to accumulate in the *p5cdh-2 *mutant. Recent results presented by Miller *et al*. (2009) suggest that ROS production by ProDH rather than P5C accumulation is the cause of the toxic effects of Pro application [22]. Unfortunately, Miller *et al*. (2009) did not analyse *ProDH *mutants and from the data presented it is not possible to determine, whether ROS production is the cause or a consequence of the loss of mitochondrial integrity that was observed in earlier studies [21]. Our identification of *pdh2 *mutants and the possibility to generate double mutants completely devoid of ProDH activity opens new possibilities to dissect the contribution of Pro degradation to Pro toxicity and stress tolerance. The detailed characterisation of the *pdh2 *mutants will also help to identify the specific physiological function of ProDH2 in the vascular tissue.

## Conclusions

Despite numerous biochemical and molecular approaches, the protective mechanism of Pro accumulation remains unclear. Each new piece of experimental evidence added to our picture of Pro metabolism in plants requires careful re-interpretation of previous results. Only by a thorough analysis of all components and also non-obvious side effects it will become possible to understand the seemingly dual nature of Pro as a protective and toxic compound. The functional characterisation of *ProDH2 *opens up a whole new set of experimental approaches to understand the function of Pro in the stress tolerance of plants.

## Methods

### Plant material and growth conditions

Arabidopsis (*Arabidopsis thaliana *(L.) Heynh. ecotype *Col-8 *or L*er*) and T-DNA or transposon insertion lines were obtained from the NASC (SALK_108179, GABI_918D08, SAIL_90_G05, GABI_328G05 (*pdh2-2*), GT1788 (*pdh2-1*), SALK_081276 (*pdh1-2*), GABI_308F08 (*pdh1-3*), SALK_119334 (*pdh1-4*) and Salk_018453 (*p5cdh-2*)), from Bernd Weisshaar (GABI_187C05; GABI-Kat program, Cologne, Germany [34], or from the KAZUSA research institute, Kisarazu, Japan (*pdh1-1*). Presence of the T-DNA and allelic status were verified by PCR and sequencing of the T-DNA flanking sequences. Gene and T-DNA-specific primers are listed in Table 1. All physiological experiments with *pdh1 *and *p5cdh *mutants were performed with homozygous progeny of plants backcrossed three times to *Col-8*. For the *pdh2-1 *mutant (in L*er *background), segregation analysis demonstrated the presence of a single transposon. Plants were cultivated axenically in 9 cm Petri dishes on commercial MS medium (Duchefa, Haarlem, Netherlands) or self-made MS medium, in which KNO_3 _and NH_4_NO_3 _were replaced by 20 mM KCl [35]. Media were supplemented with sucrose and nitrogen containing compounds as indicated for each experiment and solidified with 8 g/l purified agar (BD Biosciences, San Jose, CA, USA) when appropriate. Seeds were surface sterilised by sequential treatment with 70% (v/v) EtOH and 1% (w/v) NaOCl/0,01% (v/v) Triton-X-100 and vernalised for 24 h at 4°C in 0.1% (w/v) agarose. Plants were cultivated in a climatised room with short day (9 h) light period and a light intensity of 110 μmol photons*s^-1 ^*m^-2 ^from mixed fluorescence tubes (Biolux and Fluora, Osram, Munich, Germany) at a constant temperature of 23°C. For induction experiments, axenically grown seedlings were transferred to liquid medium and incubated in the light with agitation at 100 rpm for 6 h. Induction in liquid medium was chosen because wildtype plants metabolised proline very quickly when it was provided via the solid culture medium and only marginal increases in proline content were observed in the leaves. For seed production, plants were kept in a greenhouse with a light period of at least 16 h. A heterotrophic (white) culture was obtained from Giuseppe Forlani, Ferrara, Italy and maintained in liquid MS medium supplemented with 3% (w/v) sucrose, 1× Gamborg's vitamin mix, 0.5 mg/l 2,4-dichlorophenoxyacetic acid, 0.5 mg/l benzylaminopurine and 0,2% (v/v) Plant Preservative Medium (Plant Cell Technology, Washington, DC, USA) under constant agitation in dim light. A mixotrophic, green cell culture was obtained from Silke Robatzek (Cologne, Germany and maintained in liquid MS medium supplemented with 1% (w/v) sucrose, 4× Gamborg's vitamin mix, 0,5 mg/l 2-(1-naphthyl)acetic acid and 0,1 mg/l kinetin at 110 μmol photons*s^-1 ^*m^-2^.

**Table 1:** PCR primers used in this study

Name^1^	Sequence (5'->3')	Used for
Pdh1-LP1 (A)	tctcctctatcccaacctctg	*pdh1-1*, GABI_308F08 (*pdh1-3*), SALK_119334 (*pdh1-4*)
Pdh1-RP1 (B)	gatcgctcactcgtttcagaag	

Pdh1-LP2 (C)	aagttggtgagaggggcttac	SALK_081276 (*pdh1-2*)

Pdh1-NS-r (D)	cgcaatcccggcgattaatctc	ProDH1 cloning
Pdh1-f	caccataATGgcaacccgtcttctc^2^	

Pdh2-LP	gtaaccagcccctaaacctc	SALK_108179, GABI_918D08, SAIL_90_G05
Pdh2-RP	ggagtactagatcgcgtgtaac	

Pdh2-LP2 (F)	aaaccctaccttcgtctcac	GT1788 (*pdh2-1*), GABI_328G05 (*pdh2-2*)
Pdh2-RP2 (G)	cactaacccgttttaggacattc	

Pdh2-LP3	gagaagagttatggcttggtg	GABI_187C05
Pdh2-RP3	atgtccctttgtaatctgaattgg	

Pdh2-f (E)	caccataATGgcaaaccgtttcctc^2^	*ProDH2 *cloning
Pdh2-NSr	ccaagccataactcttctcttaag	

Pdh2-Prom-f	catttggatccttaccatccac	*ProDH2 *promoter cloning
Pdh2-Prom-r	cgggatccgtttgcCATttaaactc^2^	

Salk_LBb2	ttcggaaccaccatcaaacag	SALK lines
Gabi-LB	cccatttggacgtgaatgtagacac	GABI lines
Sail-LB2	gcttcctattatatcttcccaaattaccaataca	SAIL_90_G05
Gus5'rev	atttcacgggttggggtttc	GT1788

Put1KO-f	aaacatcgctacatagtaataacactaacgcacgcta gaaCGGATCCCCGGGTTAATTAA^3^	Put1 knockout
Put1KO-r	ttggtttgtctttgaaattggagtatatattatagtcctcG AATTCGAGCTCGTTTAAAC^3^	

SDH-f	caccacgagaataaagATGctatcgct^2^	SDH-MTP
SDH-Pdh1-r	cttgttgtccaaaggagagCTCGTGATCTATTATGTG^4^	SDH-MTP ProDH1 fusion
SDH-Pdh2-r	ggttggtcaaaggaaaggatCTCGTGATCTATTATGTG^4^	SDH-MTP ProDH2-fusion

Put1-f	caccctagaaATGatagcttcc^2^	Put1 cloning
Put1-r	taggcctactctttttggaatc	

Pdh1-pr-r	atggtcataaaacgtacttttcac	northern probes (with Pdh1-f or Pdh2-f)
Pdh2-pr-r	gactcatacacgctactcac	

P5CS1-f	aatgagaggaaaaggacaag	P5CS1 probe [8]
P5CS1-r	gataggatatgagtactaagcagag	

P5CDH-f	tggacagaagtgttctgcac	P5CDH probe
P5CDH-r	gcttccaacactagaggaag	

^1 ^Letters in brackets refer to the abbreviations in Figure 6; ^2 ^capital letters indicate the translation start codon; ^3 ^capital letters indicate the nucleotides specific for the resistance cassette, lower case letters correspond to *Put1 *5' and 3' sequences; ^4 ^capital letters correspond to the 3' sequence of the SDH-MTP, lower case letters correspond to the nucleotides encoding the N-terminus of the predicted mature Arabidopsis ProDH proteins.

### Promoter-*GUS *construct and histochemical analysis

A 1500 bp promoter fragment including the native start codon of *ProDH2 *was amplified from Arabidopsis genomic DNA with primers introducing *Bam*HI restriction sites (Tab. 1). The PCR-product was subcloned into pCR-blunt and sequenced. The promoter fragment was excised with *Bam*HI and inserted into pCB308 [36]. *Agrobacterium tumefaciens *strain GV3101 was used for floral dip transformation of Arabidopsis [37,38]. Transformants were selected by spraying soil grown seedlings with 50 mg/l BASTA or by addition of 10 mg/l BASTA to the culture medium. Histochemical GUS staining was performed according to [39].

### Expression analysis

Total RNA was extracted from snap-frozen material with phenol/guanidine thiocyanate reagent according to each manufacturer's recommendations. Per lane, 15 μg of total RNA was separated by denaturing agarose gel electrophoresis and transferred to a positively charged nylon membrane by capillary transfer. *ProDH1, ProDH2, P5CS1 *and *P5CDH *transcripts were detected by hybridisation with digoxigenin-labelled PCR products obtained with primers listed in Tab. 1, followed by detection with alkaline phosphatase coupled anti-DIG antibodies and the chemiluminescent substrate CDP-star (Roche, Basel, Switzerland). Densitometric quantification was performed with the ImageJ software. For RT-PCR, 4 μg of total RNA was converted to cDNA using random hexamer primers and the Transcriptor cDNA synthesis Kit (Roche). One μl of the cDNA preparation was used as a template for endpoint RT-PCR. Public microarray data collection were evaluated with the Expression Browser of the Bio-Array Resource and Genvestigator v3 [28,29,40,41].

### *ProDH1-GFP *and *ProDH2-GFP *constructs and imaging

The open reading frames of *ProDH1 *and *ProDH2 *without the stop codon were amplified by PCR from EST clones 38H5 and full-length ORF clone U66465, respectively (ABRC, Columbus, OH, USA). Sequences of PCR primers are given in Tab. 1. The resulting PCR fragments were purified and integrated into pENTR by directional TOPO cloning (Invitrogen, Carlsbad, CA, USA). Subsequently, the *ProDH1 *and *ProDH2 *cDNAs were transferred from pENTR to pEarleyGate103 (CD3-685, ABRC) or pGWB5 [42] by LR-recombination (Invitrogen). Both plant transformation vectors yielded the same results. Sequencing of the resulting constructs demonstrated in-frame fusion of the *ProDH *cDNAs to the *GFP *gene and revealed that EST 38H5 is not derived from Col-0 but most likely from C24 ecotype, which carries 12 silent or conservative single nucleotide polymorphisms in the *ProDH1 *coding sequence. These nucleotide exchanges are present in a number of Arabidopsis wildtype accessions. Transgenic plants were produced as described above except for selection of plants that carried pGWB5 derived constructs, which was performed in axenic culture.

Protoplasts from transformed leaves were obtained by overnight incubation with cellulase and macerase (Serva, Heidelberg, Germany), stained with MitoTracker Orange (Invitrogen) and viewed under an Olympus BX51 epifluorescence microscope equipped with a Nikon DXM1200 digital camera system (Olympus Europe, Hamburg, Germany). Chlorophyll autofluorescence, MitoTracker and GFP fluorescence of the cells were dissected using the filter sets U-MWSG2 (Olympus), 41007 and 41020 (Chroma Technology Corp, Rockingham, VT, USA), respectively. False colouring and overlay of images was performed using AxioVision software (Zeiss, Oberkochen, Germany).

### Yeast strains, growth conditions and expression plasmids

*Saccharomyces cerevisiae *strain 23344c (Matα *ura3*; [43]) was received from Giuseppe Forlani, Ferrara, Italy. For the deletion of the *Put1 *gene, the kanamycin resistance cassette from pFA6a-KanMX6 was amplified with primers adding 40 bp of *Put1 *5' and 3' sequences (Tab. 1, [44]). After transformation of 23344c with the PCR product, geneticin resistant colonies were selected and tested for their ability to utilise Pro or urea as the sole source of nitrogen. Replacement of the *Put1 *coding sequence by the resistance cassette was verified by PCR. The *Put1 *coding sequence and the *Sdh1*-mTP (bases 1-156 of the coding sequence) were amplified with the primers listed in Tab. 1 from genomic DNA isolated from 23344c. The reverse primers for the *Sdh1*-mTP introduce overlaps to the coding sequences of *ProDH1 *and *ProDH2 *that were used to fuse the coding sequences by PCR. All final PCR products were introduced into pENTR as described above and the coding sequences were transferred to pYES-Dest52 by LR-recombination (Invitrogen). Plasmids were introduced into yeast by the LiAc/PEG method [45] and transformants were selected on synthetic medium with 20 g/l glucose and 5 g/l NH_4_SO_4 _supplemented with a mixture of amino acids but no uracil (BD biosciences). For growth tests on organic nitrogen sources, yeast strains were washed once in sterile water, resuspended in 0.1% agarose and streaked on plates containing synthetic minimal medium, 20 g/l galactose and no other additives except the indicated nitrogen sources.

## Authors' contributions

GM analysed the expression pattern of ProDH2. SE generated the yeast expression constructs. DF designed the study, performed the other experiments and compiled the manuscript. All authors contributed to and approved the final manuscript.

## Supplementary Material

Additional file 1 **ProDH1-GFP is localised in mitochondria**. False colour images of a protoplast expressing a ProDH1-GFP fusion protein under control of the *CaMV*-35S promoter and stained with MitoTracker Orange. Click here for file
