# Supplementary material for: Non-redundant functions of two proline dehydrogenase isoforms in Arabidopsis
Source: BMC Plant Biol. 2010 Apr 19;10:70. doi: 10.1186/1471-2229-10-70 (PMC3095344; doi:10.1186/1471-2229-10-70)
Supplement: Additional file 1 — ProDH1-GFP is localised in mitochondria. False colour images of a protoplast expressing a ProDH1-GFP fusion protein under control of the CaMV-35S promoter and stained with MitoTracker Orange. [file 1471-2229-10-70-S1.PDF]

Additional file 1: ProDH1-GFP is localised in mitochondria

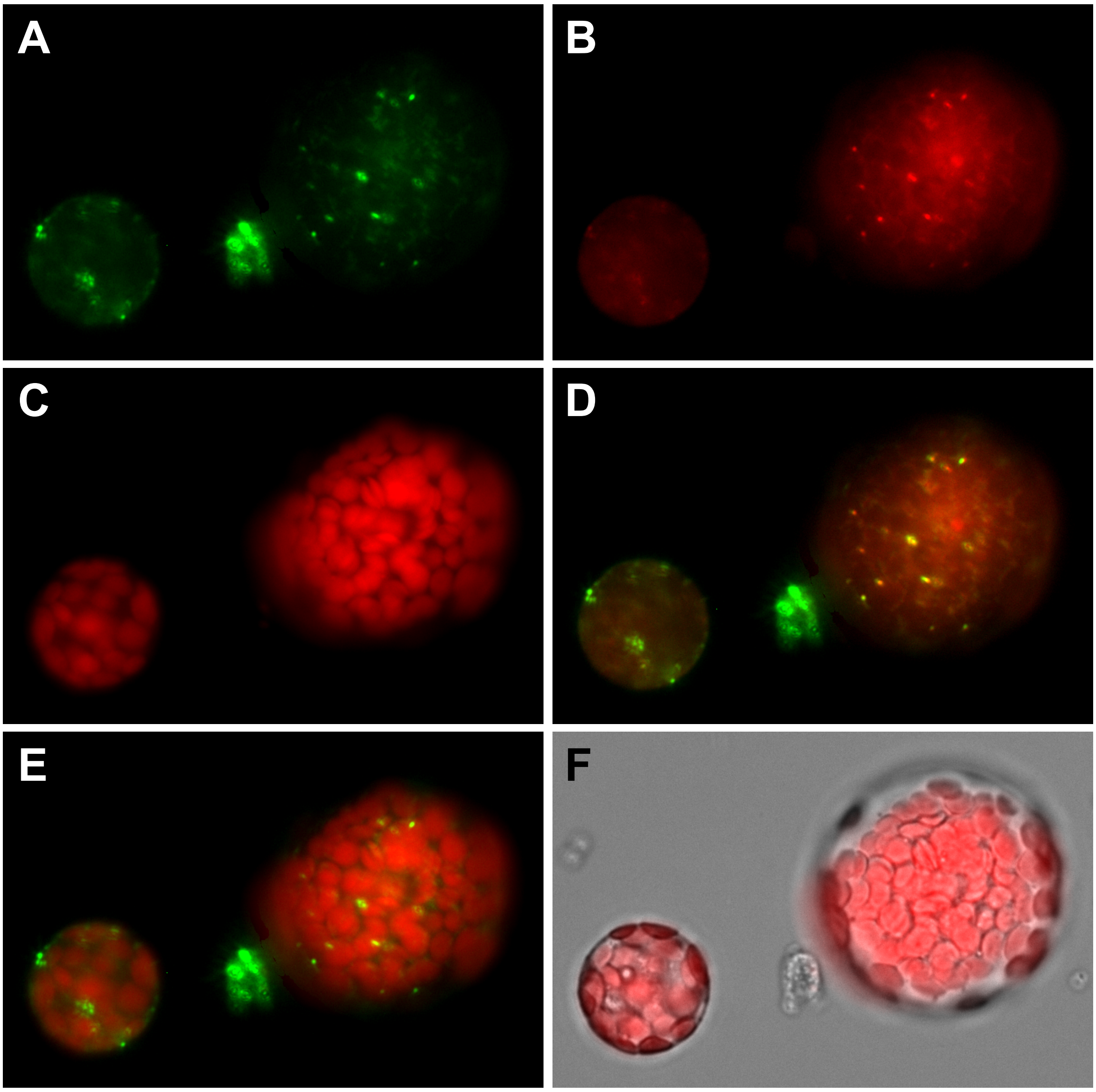

Protoplasts were isolated from mature leaves of a plant expressing a ProDH1-GFP fusion protein under control of the *CaMV* 35S promoter, stained with Mitotracker orange and viewed under an epifluorescence microscope (see material and methods section of the main text).

A: GFP fluorescence; **B**: Mitotracker fluorescence; **C**: Chlorophyll autofluorescence; **D**: Overlay of A and B; **E**: Overlay of A and C; **F**: Overlay of C with a brightfield image of the same area. Note that mitochondria in the smaller protoplast were not stained by Mitotracker. These and the green fluorescing particle between the two protoplasts demonstrate specificity of fluorescence channel separation.
